# Supplementary material for: Exportin 1 (XPO1) Expression and Effectiveness of XPO1 Inhibitor Against Canine Lymphoma Cell Lines
Source: Vet Sci. 2025 Jul 26;12(8):700. doi: 10.3390/vetsci12080700 (PMC12390048; doi:10.3390/vetsci12080700)
Supplement: Supplementary file 1 [file vetsci-12-00700-s001.zip › vetsci-3721514-supplementary.pdf]

Supplementary Materials

(a)

(b)

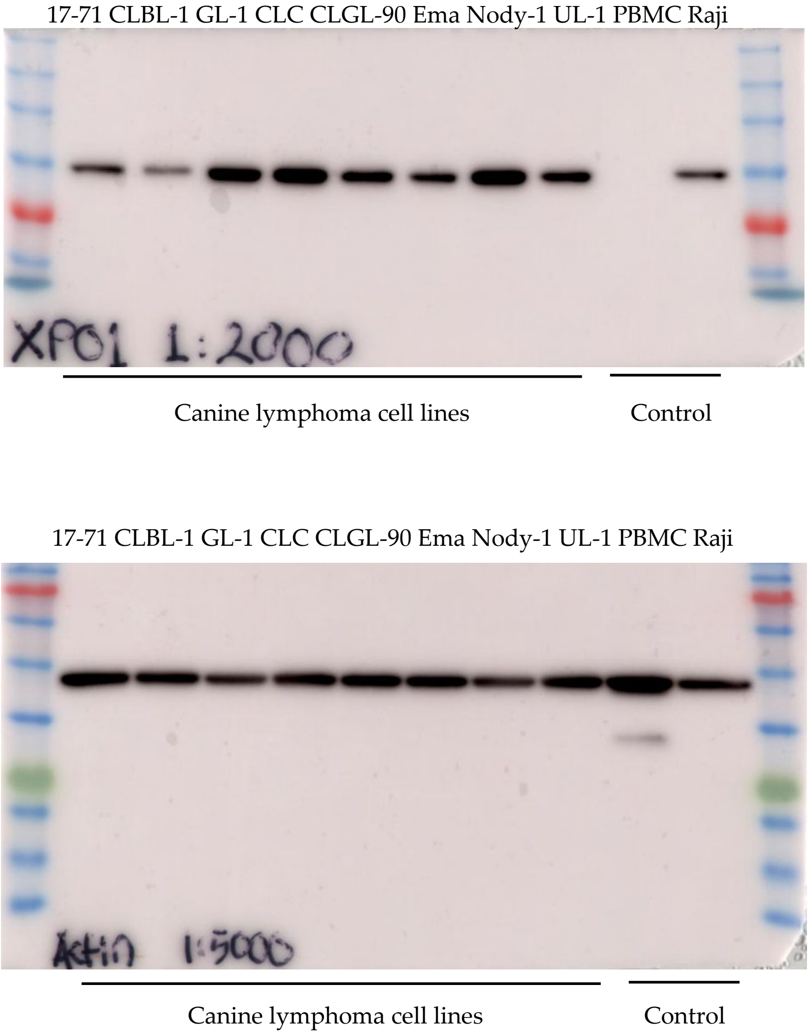

Figure S1. Original image of Western blot result in Figure 1. (**a**) The XPO1 protein expression in all canine lymphoma cell lines was performed with PBMC used as a negative control, and Raji used as a positive control. (**b**) The endogenous control for this analysis was β-actin.

Table S1. Characteristics of the eight canine lymphoma/leukemia cell lines published

Cell line	**17-71**	**CLBL-1**	**GL-1**	**CLC**	**CLGL-90**	**Ema**	**Nody-1**	**UL-1**
Classification/ diagnosis	Acute B-cell lymphoma	Multicentric B-cell lymphoma	Acute B-cell leukemia	Gastrointestinal T-cell lymphoma	Chronic large granular lymphocytic T-cell leukemia	Mediastinal T-cell lymphoma	Alimentary T-cell lymphoma	Renal T-cell lymphoma
Immunophenotype	CD1c+, CD11a+, CD11c+, CD18+, CD45+, CD45RA+, CD54+, CD49d+, CD79a+, CD34−, CD3−, TCRαβ−, TCRγδ−, CD21−	CD3−, CD4−, CD5−, CD8−, CD11a+, CD11d−, CD14−, CD21−, CD34−, CD45+, CD45RA+, CD56−, CD79αcy+, MHCII+, TCRγδ−, Thy-1−	CD3−, weak CD4+, CD5−, variably CD8+, CD11a+, weak CD11d+, variably CD14+, CD21−, CD34−, CD45+, CD45RA+, CD56−, CD79αcy+, MHCII−, TCRγδ−, Thy-1−	CD3−, CD4−, CD8α−, CD11a−, CD11b−, CD11c−, CD14−, CD18+, CD21−, CD34−, CD45+, CD45RA+, CD90−, TCRαβ−, TCRγδ−, MHCII+, IgM−, IgG−	CD45R, CD18+, CD3+, TCRαβ+, variably CD8α+, CD79a−	CD3+, CD4−, CD8α−, CD11a−, CD11b−, CD11c−, CD14−, CD18+, CD21−, CD34−, CD45+, CD45RA+, CD90+, TCRαβ−, TCRγδ+, MHCII−, IgM−, IgG−	CD3+, CD4−, CD8α−, CD11a−, CD11b−, CD11c−, CD14−, CD18+, CD21−, CD34−, CD45+, CD45RA+, CD90−, TCRαβ−, TCRγδ−, MHCII+, IgM−, IgG−	CD3−, CD4−, CD8α+, CD11a−, CD11b−, CD11c−, CD14−, CD18−, CD21−, CD34−, CD45+, CD45RA+, CD90−, TCRαβ−, TCRγδ−, MHCII−, IgM−, IgG−
PARR	ND	TCRγ−, IgH+	TCRγ+, IgH−	TCRγ−, IgH−	ND	TCRγ+, IgH−	TCRγ+, IgH−	TCRγ+, IgH−
p53/NFκB protein expression	ND	+	+	ND	ND	−	−	−
p16 protein expression	+	−	+	−	−	−	−	−
Phospho-pRb protein expression	−	+	−	+	+	+	+	+
ABCB1 (*MDR1*) gene expression	ND	Low	Low	ND	ND	high	ND	high
Initial description or publication	[18,38-40]	[18,41-45]	[18,40-46]	[18,42,47]	[18,39,40]	[18,42,44,45,47]	[18,42,44,47]	[18,42,44,45,47,48]

ND= Not Determined
